# Closed-Loop Acoustic Stimulation Enhances Sleep Oscillations But Not Memory Performance

**DOI:** 10.1523/ENEURO.0306-19.2019

**Published:** 2019-11-05

**Authors:** Simon Henin, Helen Borges, Anita Shankar, Cansu Sarac, Lucia Melloni, Daniel Friedman, Adeen Flinker, Lucas C. Parra, Gyorgy Buzsaki, Orrin Devinsky, Anli Liu

**Affiliations:** 1 NYU Comprehensive Epilepsy Center, New York, New York 10016; 2 New York University Langone Health, New York, NY 10016; 3 Max Planck Institute for Empirical Aesthetics, 60322 Frankfurt am Main, Germany; 4 Department of Biomedical Engineering, City College of New York, New York, New York 10031; 5 NYU Neuroscience Institute, New York, New York 10016

**Keywords:** acoustic stimulation, declarative memory, memory, oscillations, sleep, spindles

## Abstract

Slow oscillations and spindle activity during non-rapid eye movement sleep have been implicated in memory consolidation. Closed-loop acoustic stimulation has previously been shown to enhance slow oscillations and spindle activity during sleep and improve verbal associative memory. We assessed the effect of closed-loop acoustic stimulation during a daytime nap on a virtual reality spatial navigation task in 12 healthy human subjects in a randomized within-subject crossover design. We show robust enhancement of slow oscillation and spindle activity during sleep. However, no effects on behavioral performance were observed when comparing real versus sham stimulation. To explore whether memory enhancement effects were task specific and dependent on nocturnal sleep, in a second experiment with 19 healthy subjects, we aimed to replicate a previous study that used closed-loop acoustic stimulation to enhance memory for word pairs. The methods used were as close as possible to those used in the original study, except that we used a double-blind protocol, in which both subject and experimenter were unaware of the test condition. Again, we successfully enhanced slow oscillation and spindle power, but again did not strengthen associative memory performance with stimulation. We conclude that enhancement of sleep oscillations may be insufficient to enhance memory performance in spatial navigation or verbal association tasks, and provide possible explanations for lack of behavioral replication.

## Significance Statement

Prior studies have demonstrated that a closed-loop acoustic pulse paradigm during sleep can enhance verbal memory performance. This technique has widespread scientific and clinical appeal due to its noninvasive nature and ease of application. We tested with a rigorous double-blind design whether this technique could enhance key sleep rhythms associated with sleep-dependent memory performance. We discovered that we could reliably enhance slow and spindle rhythms, but did not improve memory performance in the stimulation condition compared with sham condition. Our findings suggest that enhancing slow-spindle rhythms is insufficient to enhance sleep-dependent learning.

## Introduction

Numerous behavioral studies have demonstrated the benefit of sleep for stabilizing memories (Rasch and Born, 2013). Proposed mechanisms include the passive protection of newly acquired memories from interference (Jenkins and Dallenbach, 1924), as well as active systems consolidation through repeated reactivation of newly acquired memory traces, supported by hippocampal sharp-wave ripples (Buzsaki, 1989, 2015). Non-rapid eye movement (NREM) sleep is considered important in the latter process, when labile memory traces in the hippocampus are distributed to long-term storage sites in neocortex (Laureys et al., 2001). Memories become integrated with older information, becoming qualitatively transformed in the process (Diekelmann and Born, 2010; Dudai et al., 2015). In addition, sleep may downscale synapses globally (Tononi and Cirelli, 2019).

**Figure 1.**
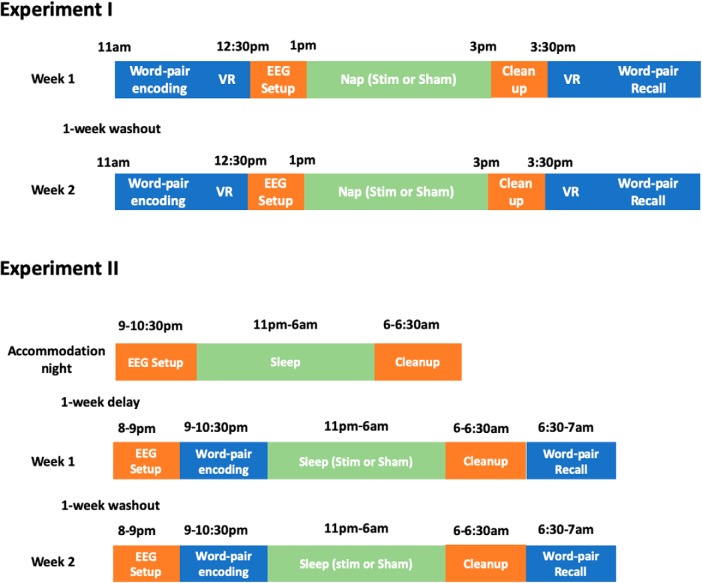
Schema for experiments 1 and 2. Timing of different phases for each experiment. In experiment 1, subjects arrived at the laboratory at 11:00 A.M. Following informed consent, participants performed the encoding portion of the word pair associates and virtual reality (VR) tasks, followed by setup of EEG electrodes. After a 2 h nap opportunity, participants completed the recall portions of the VR and word pair associates tasks, respectively. In experiment 2, participants arrived at the sleep laboratory at 9:00 P.M. for the accommodation night (no memory tasks or stimulation performed). On experimental nights (weeks 1 and 2), participants arrived at the sleep laboratory at 8:00 P.M. for EEG electrode setup, followed by word pair associates encoding at ∼9:00 P.M. Light’s out started at 11:00 P.M., and participants were awoken at ∼6:00 A.M. (e.g., after 7 h). A 1 week delay (washout) occurred between every session.

NREM sleep is accompanied by recurrent events in the local field potential that contribute to memory consolidation. Multiscale unit recordings in rodents demonstrate that coupling between neocortex and hippocampus is synchronized on both coarse (1–2 s) and fine-grained (10 ms) temporal scales during sleep, presumably to coordinate spike timing-dependent information transfer between the two structures (Sirota et al., 2003; Isomura et al., 2006; Fig. 1). The depolarizing neocortical “UP state” of slow oscillations (SO; ∼0.5–4 Hz) drives thalamocortical spindles (transient 10–18 Hz oscillations; Steriade, 2006). Intracranial recordings in humans demonstrate that cortical DOWN states coincide with hyperpolarization of thalamic neurons, followed by thalamocortical spindles (Steriade et al., 2001; Mak-McCully et al., 2017; Gonzalez et al., 2018). In turn, spindle troughs group hippocampal sharp-wave ripples (SPW-Rs). SPW-Rs emerge in the recurrent excitatory system of CA2-CA3 hippocampal neurons and their population synchrony depolarizes CA1 neurons. In turn, the interactions between CA1 pyramidal cells and perisomatic inhibitory interneurons bring about a transient high-frequency oscillation (100–180 Hz; i.e., the ripple) in CA1 pyramidal layer (Sirota et al., 2003; Steriade, 2006). SPW-Rs replay time-compressed fragments of spike trains observed during the experiences of the previous day and are critical for the consolidation of these experiences (Wilson and McNaughton, 1994; Diba and Buzsáki, 2007; Girardeau et al., 2009).

Several experiments suggest the critical role of SO spindle complexes in memory consolidation. In humans, spindles are reactivated during sleep in the cortical regions that were involved in learning (Bergmann et al., 2012; Cox et al., 2014a). Specific reactivations of information learned during the prior day (e.g., discrimination between faces and houses) can be decoded from spindles detected from scalp EEG recordings (Schönauer et al., 2017). Suppressing spindles can disrupt the benefits of sleep on memory (Schreiner et al., 2015). Feedback-controlled 12 Hz transcranial alternating current stimulation (stim) has been reported to selectively enhance spindle incidence during NREM sleep and related consolidation of learned sequences of motor actions (Lustenberger et al., 2016). In mice, when applying closed-loop optogenic stimulation of the thalamic reticular nucleus, only spindles induced during the UP state augmented coupling between SO spindle ripples and hippocampus-based memory. In contrast, spindles occurring out of phase with the UP state did not enhance memory in a contextual fear paradigm (Latchoumane et al., 2017).

Manipulating the cortico-thalamo-cortical coupling of SO spindle complexes offers an appealing opportunity to indirectly increase the incidence of SPW-R events in a safe and accessible manner. Attempts to enhance these coupled rhythms have used various stimulation methods in humans, including transcranial electrical stimulation, transcranial magnetic stimulation, and acoustic stimulation (Marshall et al., 2006; Massimini et al., 2007; Ngo et al., 2013). Closed-loop acoustic stimulation has been applied in various patterns, including closed-loop single pulse, closed-loop paired pulse, and blocks of five 1 Hz pulses to potentially augment SO spindle oscillations and associated declarative memory (Ngo et al., 2013; Ong et al., 2016; Leminen et al., 2017; Papalambros et al., 2017).

Using invasive electroencephalography, we found that acoustic stimulation can enhance slow-spindle activity in widespread brain regions (Lafon et al., 2017). Encouraged by these robust physiologic results, we performed two experiments testing the behavioral effects of closed-loop acoustic stimulation on two hippocampus-dependent memory tasks.

In the first experiment, we tested whether closed-loop acoustic stimulation applied during short daytime sleep could enhance performance on a visuospatial navigation task (Miller et al., 2013). We found that stimulation reliably enhanced SO spindles but not memory performance. Given these results, we decided to revisit a previous study applying closed-loop acoustic stimulation during night-time sleep, which resulted in improved memory for word associations (Ngo et al., 2013). We followed the original experimental protocol, except now blinding the experimenter in addition to the subject. Again, we replicated the enhancement of SO spindles during NREM sleep but did not find an improvement in verbal associative memory. We offer possible explanations for how SO spindle activity could be elicited without associated behavioral changes.

## Materials and Methods

### Participants

Subjects were recruited primarily from a recruitment website hosted by the NYU Department of Psychology between May 2017 and March 2019. Inclusion criteria consisted of the following: (1) fluent English speakers; (2) age 18–30 years; and (3) able to provide informed consent. Subjects were excluded if (1) they had received a diagnosis of any neurologic or psychiatric disorder (except for migraine headache), (2) had any sleep disorder (e.g., insomnia, sleep apnea, delayed circadian cycle, parasomnia, restless legs), or (3) had used psychoactive medication (e.g., antidepressants, anxiolytics, stimulants) or (4) alcohol or recreational drugs in the 24 h before the study; (5) had experienced a migraine headache in the 48 h before the study; (6) had a body mass index >30 (increasing the risk for sleep apnea); (7) had recently traveled across time zones within the month before participation; (8) had engaged in shift work for 1 month before participation; and (9) had scored <26 of 30 on the Montreal Cognitive Assessment (MOCA). On the day of each session, participants were instructed to wake up an hour earlier than usual, to refrain from caffeine consumption, and not to take any naps before the experiment. The study was approved by the institutional review board by the NYU School of Medicine, and all subjects gave written consent before participation.

A total of 31 subjects participated in the study (mean age, 23.5 ± 0.6 years), composed of two experiments (Fig. 1). Twelve subjects completed experiment 1 (mean age, 23.3 ± 2.7 years; mean length of education, 15.8 ± 1.2 years), including an afternoon stimulation nap and sham nap sessions separated by at least 1 week. Based on previously reported effect sizes (Ngo et al., 2013), the experiment 1 study achieved a statistical power of 92% to detect a moderate effect size (Cohen’s *d* = 0.5). Nineteen subjects completed experiment 2, including three overnight sleep sessions (an accommodation night, stimulation condition, and sham condition) separated by at least 1 week. Subjects were 47.4% female (9 of 19 subjects), a mean of 23.3 years (SD, 3.4 years) of age, with a mean of 14.7 years (SD, 1.5 years) of education. Based on previously reported effect sizes (Ngo et al., 2013), experiment 2 achieved a statistical power of 95% to detect a moderate effect size. Subject demographic characteristics are presented in Table 1.

**Table 1.** Subject characteristics

	Experiment 1 (*N* = 12)		Experiment 2 (*N* = 19)	
Female (*n*, %)	6.0	50.0%	9.0	47.4%
Age, years (mean, SEM)	23.3	0.8	23.3	0.9
Race (*n*, %)				
Caucasian/white	5.0	41.7%	8.0	42.1%
African American/black	4.0	33.3%	2.0	10.5%
Asian	3.0	25.0%	5.0	26.3%
Biracial	0.0	0.0%	1.0	5.3%
Not specified	0.0	0.0%	3.0	15.8%
Hispanic/Latino (*n*, %)	0.0	0.0%	2.0	10.5%
Education, years (mean, SEM)	15.8	0.3	14.7	0.4
SSS score visit 1 maximum score = 6				
Pre (mean, SEM)	2.9	0.3	3.5	0.4
Post (mean, SEM)	2.2	0.3	2.6	0.2
SSS score visit 2 maximum score = 6				
Pre (mean, SEM)	2.3	0.2	3.5	0.3
Post (mean, SEM)	2.2	0.3	2.5	0.3
PVT score visit 1				
Pre (mean, SEM)			0.3	0.0
Post (mean, SEM)			0.3	0.0
PVT score visit 2				
Pre (mean, SEM)			0.3	0.0
Post (mean, SEM)			0.3	0.0
MOCA score maximum score = 30				
(mean, SEM)	27.9	0.4	28.6	0.3

Demographics table of subjects who participated in experiments 1 and 2.

### Electroencephalographic recording

For experiment 1, electroencephalogram (EEG) recordings were recorded continuously using a NeuroConn DC amplifier sampled at 128 Hz. A limited polysomnographic montage using the international 10-20 system (electrodes F3, Fz, F4, C3, C4, P3, Pz, P4, O1, and O2; vertical electro-oculogram, horizontal electro-oculogram, and 2× EMG) with a mastoid reference using gold-cup electrodes were used. The ground electrode was placed on the vertex. Impedances were kept at <5 kΩ. For experiment 2, an EEG was recorded using the BrainVision LiveAmp DC amplifier sampled at 250 Hz. A full 20/10 EEG montage was used (Fp1, Fp2, F7, F3, Fz, F4, F8, T3, C3, Cz, C4, T4, T5, P3, Pz, P4, T6, O1, and O2) referenced to the linked mastoids using gold-cup electrodes, with ground placed near the vertex. Real-time signals were filtered between 0.3 and 50 Hz, and stored to disk. An additional trigger channel was used to mark detections of slow waves and audio delivery for off-line analysis.

### Closed-loop acoustic stimulation

Closed-loop stimulation aimed to deliver auditory pulses (50 ms pink noise, 5 ms on/off ramp) targeted to the UP state of ongoing slow-waves (UP states). Acoustic stimulation was delivered using flat-profile headphones intended for sleep (Dubslabs), and the volume was adjusted between 50 and 65 dB SPL before sleep to facilitate patient comfort. Real-time EEG analysis used published protocols (Ngo et al., 2013). Briefly, stimulation (or sham) was started once stable NREM stage 2 or 3 sleep was observed for ≥2 min. During this period, slow-wave DOWN states were detected from the electrode Fz signal, low-pass filtered between 0.5 and 4Hz. Slow-wave DOWN states were detected if the voltage exceeded the initial threshold of −80 μV. To account for changes in overall increases/decreases in slow-wave amplitude over time, the threshold was updated every 2 s to minimum voltage in the previous 2 s or −80 μV.

In experiment 1, a single auditory pulse was delivered 0.5 s after detection of a slow-wave DOWN state (equivalent to 1 Hz slow-oscillation frequency). Preliminary pilot experiments determined that single-pulse 1 Hz stimulation could produce robust slow-wave entrainment and has been used in a published paradigm (Leminen et al., 2017). After each slow-wave detection, detection was paused for 3 s after delivery of the acoustic pulse to allow for recovery from a refractory period after stimulation (Ngo et al., 2015). During sham stimulation, the same procedure was followed except that the volume was disabled.

Experiment 2 followed a similar procedure, except that the stimulation frequency was predetermined for each subject based on the individual slow-wave frequency estimated from their accommodation night (Ngo et al., 2013). Additionally, a paired acoustic pulse paradigm was used, where a second auditory pulse was delivered 1.075 s after the first pulse. After each detection of a slow-wave event, detection was paused for 2 s after delivery of the second acoustic pulse. The detection algorithm was activated throughout the 210 min sleep period but paused whenever the subject left NREM sleep or woke up, as determined by live monitoring of the sleep EEG. Triggers marking stimulation delivery were saved in the EEG data for later event-related analysis. During sham stimulation, the same procedure was followed except that the volume was disabled.

### Study design and procedures

#### Experiment 1

##### Closed-loop acoustic stimulation during daytime nap

Using a within-subjects crossover design, subjects received either acoustic or sham stimulation in one of two test daytime sessions, separated by at least 1 week. Participants were instructed to arrive at the laboratory at 11:00 A.M., where they were consented for participation and then prepared for EEG recording. Immediately after the EEG setup, participants completed the Stanford Sleepiness Scale (SSS), followed by two hippocampally mediated memory tasks: (1) a word pairs associates task; and (2) a spatial navigation task. Following these behavioral assessments, participants were given a 2 h nap opportunity. On waking from the nap, participants were given the postsleep memory assessment, described below.

##### Memory tasks

Two memory tests investigated whether acoustic stimulation differentially affects two kinds of hippocampally mediated memory, as follows. (1) In the word pair association, participants were shown the following instructions: “This experiment will ask you to memorize 100 word pairs. You will be tested immediately after seeing all the pairs, and for a second time when you wake up from the nap.” Participants were then presented with 100 unrelated word pairs displayed serially on a computer screen [4 s stimulus interval, 1 s interstimulus interval (ISI)]. Words of moderate concreteness level were selected from the University of Western Australia School of Psychology MRC Psycholinguistic Database (Coltheart, 1981). Next, in the cued recall phase, only the first word in a pair was shown and the subject was asked to recall the associated word. No reinforcement or feedback was given. Participants repeated this recall phase 30 min after waking from their nap. The word pair sequence was randomized across presentations and participants. For each test session, a different set of 100 word pairs was presented. Word lists were randomized across conditions. Performance on this task was assessed by taking the difference in the number of word pairs remembered postnap versus prenap (Retention). (2) In the spatial navigation task, we used a virtual reality (VR) spatial navigation task developed to probe hippocampus-dependent spatial memory performance (Miller et al., 2013). Participants played a computer-based video game in which the keyboard is used (up/down/left/right arrow keys) to ride a bike around a virtual city that contained storefronts (e.g., grocery store, bakery). During an initial training phase, participants freely navigated the city and were asked to find stores within the city (e.g., “Find the grocery store”). Training was completed once each store had been visited three times. During the test phase, the player was placed at predefined locations within the virtual city, and asked to navigate to 1 of 11 stores as quickly as possible. After napping, participants completed a second test phase, with the order of store visits randomized. Two virtual city layouts (same city plan, different storefront locations) were generated and randomly assigned to each visit (control vs treatment). Participants were shown the following instructions: “Use the arrow keys to navigate to the store indicated in the upper left corner. When you arrive at the store, you will hear the name of the object you just delivered. Try to remember it and the store together as a pair, then travel to the next store. You will make 16 deliveries. After you are done, you will be asked to say out loud all the objects you can remember in any order. Then you will be shown the stores and asked to remember which object you delivered to each. Do you have any questions? Please ask the facilitator. Then, press ‘x’ to start the task.” Performance on this task was assessed by tabulating the number of postnap speed improvements (Speed Improvement) for each storefront (e.g., the difference in time taken to reach each storefront after the nap vs before the nap). During each behavioral testing session, general vigilance and subjective sleepiness was assessed using the SSS (Hoddes, 1972). In this cohort (*N* = 12), the SSS did not show a statistically significant difference in sleepiness between the stimulation and sham conditions (*F*_(1,11)_ = 1.54, *p* = 0.24).

#### Experiment 2

##### Closed-loop acoustic stimulation during overnight sleep

We next performed a replication of the original published study (Ngo et al., 2013), which has shown positive effects on declarative memory and has been replicated by several groups, although with smaller effect sizes (Ong et al., 2016; Leminen et al., 2017; Papalambros et al., 2017). The only procedural differences in our study were that (1) the original German word pairs were translated into English, and (2) ours was a double-blind study, in which both the subjects and the tester were unaware of the test condition. We included 19 subjects, which increased the statistical power of our findings, compared with previous studies.

As in the original study, testing was performed over three overnight sleep sessions (accommodation, sham, and treatment nights), separated by at least 1 week. Participants were instructed to arrive at 8:00 P.M. at the sleep facility. After EEG and polysomnographic preparation, a word pair association task was presented to the subject, and the subject was instructed to fall asleep. EEG recordings began at 11:00 P.M. (lights out) and continued until ∼7:00 A.M. (lights on). Thirty minutes after awakening, participants performed postsleep memory assessments. Auditory stimulation started ∼5 min after the subject displayed stable stage 2 sleep. The stimulation continued for 210 min, was stopped if the subject aroused, and continued only after the subject fell back to NREM stage 2 sleep. The first night was an accommodation night to ensure that participants could sleep and to determine the individual slow-wave oscillation latency. The second and third nights were experimental nights, when either real or sham acoustic stimulation was delivered. Conditions were assigned in a randomized and counterbalanced order.

##### Memory tasks

We used a declarative memory task of 120 moderately semantically related word pairs (e.g., brain - consciousness), which were translated from the original German lists (Ngo et al., 2013, 2019) into English. A different word list was used between the two experimental sessions. Participants were shown the following instructions: “This experiment will ask you to memorize 120 word pairs. You will be tested immediately after seeing all the pairs, and for a second time when you wake up in the morning.” The subjects were then shown the 120 word pairs serially (4 s stimulus presentation, 1 s ISI) via a laptop computer screen. This was followed by an immediate recall session in which participants were shown the first word in the pair and asked to freely recall the associated word. Subjects had unlimited time to respond. In contrast to experiment 1, after being given the opportunity to provide the paired word, the correct answer was shown to provide an additional encoding opportunity. On waking in the morning, participants completed another recall session (postsleep recall), in which they were shown the same 120 initial words (but in a different order), and asked to recall the paired word (but without feedback). Participants were shown the following instructions: “You will be shown a word from the pairs of words you saw last night. Please say aloud the word that it was paired with.” Performance on this task was assessed by subtracting the number of word pairs remembered in the morning from the number of correctly remembered words the evening before. To assess for the influence of arousal and executive functioning, after memory testing, participants performed the psychomotor vigilance task (PVT) and completed the SSS. In the PVT, for a 5 min interval a counter appeared in the middle of the screen every 2–10 s, and subjects were instructed to stop the counter as quickly as possible with a keystroke. In this cohort (*N* = 19), no significant differences in sleepiness or alertness were observed between the stimulation and sham conditions (SSS: *F*_(1,18)_ = 2.89, *p* = 0.11; PVT: *F*_(1,18)_ = 0.03, *p* = 0.86).

### Off-line EEG analysis

For both experiments, off-line sleep staging was performed on 30 s EEG epochs using a combination of a custom, automated sleep**-**staging algorithm followed by a blinded review by two raters (A.L., S.H.) according to standard criteria (Berry et al., 2012). For each sleep session, total sleep time (TST), and time spent in each stage N1, N2, N3 [slow-wave sleep (SWS)], REM, and wakefulness was determined as an absolute number of minutes and a percentage of TST. Differences between the time spent in each stage of sleep and TST per condition (treatment vs sham) were determined by paired *t* tests.

Analysis of the acute physiologic effects of stimulation was performed by computing time-locked averages to the first auditory pulse, or the corresponding time point in the sham stimulation condition. Recorded EEG was referenced to the linked mastoids. Off-line detection of all SOs during the whole overnight recording period was performed following a previously described method (Ngo et al., 2013). A virtual channel was constructed from the mean of a subset of EEG electrodes (F3, Fz, F4, C3, Cz, C4, P3, Pz, and P4), and SOs were identified via zero-crossing detection as events whose through-to-peak amplitude exceeded 1.25× the average across both stim and sham conditions, exceeded 1.25× the average negative peak across conditions, and with a duration between 0.9 and 2 s (0.5–1.11 Hz). Subsequently, all SOs detected within all NREM segments were time locked to the peak of the negative DOWN state. Analysis of sleep spindles was performed by filtering the raw EEG trace in the slow-spindle (9–12 Hz) and fast-spindle (12–15 Hz) frequency bands, and computing the root mean square amplitude of the filtered response in 100 ms windows. Spindle amplitude was then time locked to the DOWN states of the previously identified SO events.

### Statistical analysis

All data are presented as the mean (±SEM). In order not to bias our results, we performed the same statistical testing procedures used in the original study (Ngo et al., 2013). All statistical analyses were performed using MATLAB (R2018b, MathWorks) using paired-sample *t* tests. A two-way repeated-measures ANOVA was conducted that examined the effect of treatment type (sham vs stimulation) and time (evening vs morning) on SSS and PVT scores. A *p* value <0.05 was considered significant. To assess the possibility of null effects, additional Bayesian statistics were performed using JASP (http://jasp-stats.org). Analysis of effect sizes and statistical power were performed using Hedges *g*_av_, using the average SD across conditions, and corrected for the number of paired samples (Lakens, 2013).

## Results

### Closed-loop acoustic stimulation during daytime sleep enhances slow oscillations and spindles but not spatial navigation performance

For experiment 1, EEG recordings from the 12 subjects who completed stimulation and sham sessions were examined to determine whether acoustic stimulation, when delivered during the UP state of ongoing slow-wave oscillations, entrains the underlying oscillations (SOs and sleep spindles). Slow waves were analyzed by comparing averaged responses, time locked to the acoustic or sham stimulation. In the stimulation condition, with real-time detection of the SO negative half-wave peak during NREM sleep, one auditory pulse (50 ms, pink noise) was delivered, timed to the subsequent SO UP state. Acoustic stimulation started within 5 min of NREM sleep onset and was discontinued after the 2 h nap or when the subjects aroused from nap sleep. Stimulation was delivered for a mean ± SEM of 58.7 ± 9.1 min. Subjects napped an average ± SEM of 83.0 ± 9.6 min in the stimulation condition, and 79.3 ± 7.31 min in the sham condition (*t*_(11)_ = −0.41, *p* = 0.69). Subjects spent a similar amount of time in each stage of sleep; stimulation did not impair sleep quality or duration (Table 2).

**Table 2.** Mean time spent in each sleep stage during experiment 1 (nap study)

	Stim	Sham	*p* Value
W	7.42% (1.85)	6.29% (1.73)	0.49
N1	20.92% (2.96)	20.92% (1.59)	0.53
N2	30.77% (4.04)	33.26% (4.19)	0.57
SWS	16.53% (2.73)	18.45% (2.03)	0.40
REM	22.57% (6.91)	21.01% (5.16)	0.78
MA	0.08% (0.08)	0.07% (0.06)	0.92
TST (mins)	83.0 (9.60)	79.3 (7.31)	0.69

Sleep during daytime nap study is characterized with the average time spent in each sleep stage (mean ± SEM). The percentage of time spent in each condition (W, wake; N1, stage 1; N2, stage 2; MA, movement artifact) was similar during the stimulation period, demonstrating that stimulation did not disrupt sleep or increase the overall time spent in non-REM sleep.

**Figure 2.**
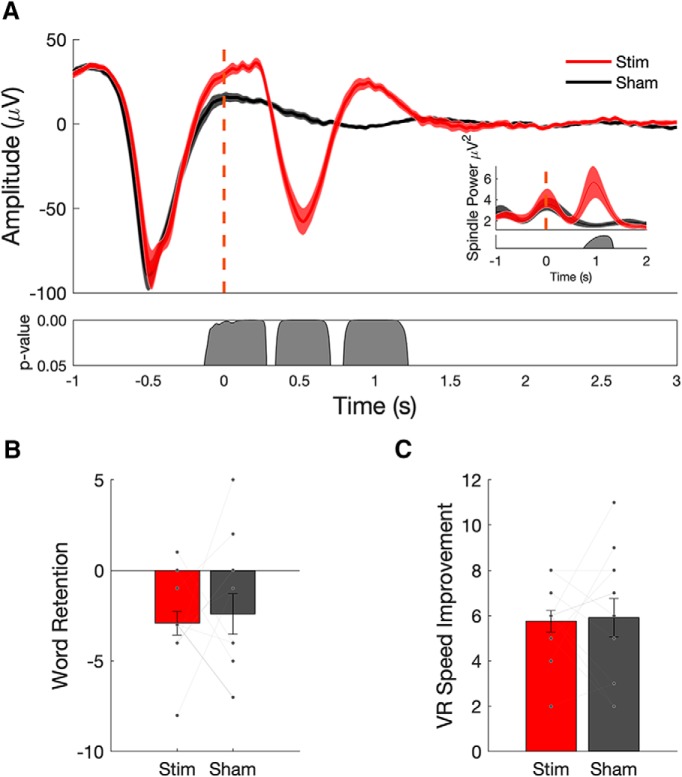
Closed-loop stimulation during a daytime nap enhances slow wave–spindle complexes, but does not enhance memory performance. ***A***, Evoked responses from 12 subjects (mean ± SEM) shows that stimulation delivered during slow-wave UP states (red curves; dashed red line indicates the onset of acoustic pulse) enhances ongoing slow-wave oscillations (red) compared with sham stimulation (black). Spindle power is also increased in stimulation (red) compared with sham (black; inset). ***B***, ***C***, Memory performance, as assessed by postnap retention of word pairs (***B***), and spatial navigation performance, as measured by the number of speed improvements (***C***), do not exhibit a significant benefit from acoustic stimulation relative to sham (each line represents individual subject performance).

Averaging the EEG time locked to the acoustic pulse demonstrated an increase in SO activity compared with the sham condition (Fig. 2*A*). The stimulation condition induced a second SO cycle after the endogenous cycle that triggered the stimulation, whereas the sham condition only exhibits the endogenous SO cycle. Spindle power (12–16 Hz) increased during single-pulse acoustic stimulation (Fig. 2*A*, inset) at ∼1 s after stimulation, coinciding with the UP state of the induced second SO cycle. Off-line detection revealed no difference in the total number, amplitude, or slope of SO events (Table 3). However, a shift in SO frequency toward 1 Hz (i.e., to a slightly shorter SO duration; 1.13 ± 0.01 vs 1.15 ± 0.01 s) was observed in the stimulation compared with sham condition (Table 3), which is likely the result of the stimulus-induced SO.

**Table 3.** Slow-oscillation characteristics during afternoon nap closed-loop stimulation (experiment 1)

	Stim	Sham	*p* Value
Number of SOs	166.67 (27.76)	154.58 (17.65)	0.52
SO amplitude (μV)	147.36 (13.04)	143.17 (9.89)	0.40
SO slope (μV/s)	278.68 (26.09)	275.92 (20.30)	0.83
Duration (s)	1.13 (0.01)	1.15 (0.01)	0.04

Mean ± SEM number of slow oscillations (identified off-line; see Materials and Methods) during the entire recording, amplitude (negative half-wave-to-peak), slope, and duration between stim and sham conditions. Duration of SO in the stimulation condition was significantly shorter compared with the sham condition, suggesting that the induced SO oscillation peaked at a higher frequency compared with the sham condition (∼1 Hz)

Memory performance measured as the number of items that showed a speed improvement through the virtual environment (Fig. 2*C*; *t*_(11)_ *=* −0.1881, *p* = 0.85) or performance on a verbal associative memory task (Fig. 2*B*; *t*_(11)_ = −0.3490, *p* = 0.73) did not show a significant benefit from stimulation when compared with sham. Subjects did not perform at ceiling or floor, averaging between 45% and 48% of word pairs recalled on pre-nap and post-nap recall tests. In addition to parametric statistics, we also performed additional Bayesian statistics to support the null hypothesis that memory performance across the conditions did not differ (see Table 4).

**Table 4.** Bayesian paired-samples *t* test for memory tests in experiments 1 and 2

	BF_10_	Error %
Experiment 1		
Word pair retention		
Stim-sham	0.302	0.019
VR speed improvements		
Stim-sham	0.299	0.019
Experiment 2		
Word pair retention		
Stim-sham	0.249	0.012

Bayes factor (BF_10_) and proportional error of the BF for paired-samples *t* test of the hypothesis the memory performance scores in the stim and sham sessions are equal. A Bayes factor <1 indicates evidence in favor of the null hypothesis (e.g., stim = sham)

**Figure 3.**
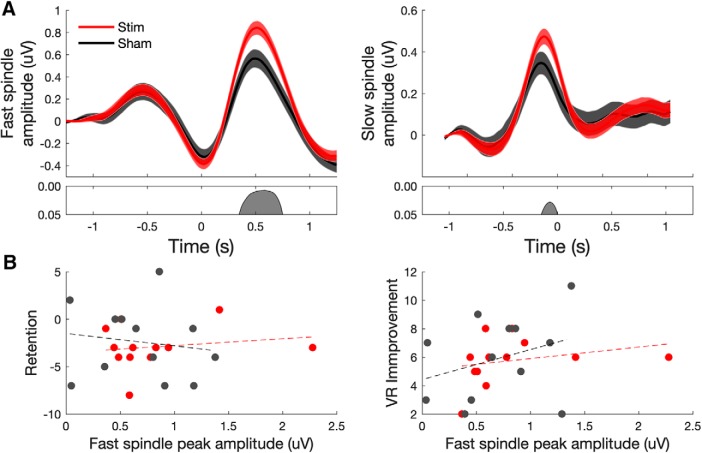
Acoustic stimulation during nap sleep enhances both fast- and slow-spindle amplitudes but is not related to memory performance. ***A***, Fast-spindle (12–15 Hz) and slow-spindle (9–12 Hz) amplitude at electrode Cz time locked to the negative DOWN state of all off-line detected SO events (*t* = 0). Both fast- and slow-spindle amplitude showed significant increases in amplitude in the stimulation (red) condition relative to sham (black) stimulation. ***B***, Scatter plots of retention versus peak fast-spindle amplitude (left) and the number of VR improvements versus peak fast-spindle amplitude (right) across individuals in sham and stimulation conditions (no significant correlations in either task).

An analysis of both fast- and slow-spindle band amplitude relative to all off-line detected SOs (see Materials and Methods) showed an increase in both fast- and slow-spindle band amplitude in the stimulation condition relative to sham stimulation (Fig. 3*A*). However, no significant correlations were found between peak fast-spindle amplitude and memory performance in either condition or memory task (Fig. 3*B*).

### Closed-loop acoustic stimulation during overnight sleep enhances slow-spindle activity but not verbal memory performance

While a robust physiologic effect of closed-loop acoustic stimulation was observed in experiment 1, we did not observe any measurable benefit to declarative or spatial memory performance. Because a previous nap study demonstrated only a modest memory benefit with stimulation (Ong et al., 2016), we questioned whether the effect size was too small to be measured with nap sleep but could be demonstrated with overnight sleep. We also considered whether differences in the behavioral paradigm contributed to the reported memory improvement in the previous experiments and thus replicated the original study as faithfully as possible (Ngo et al., 2013).

**Figure 4.**
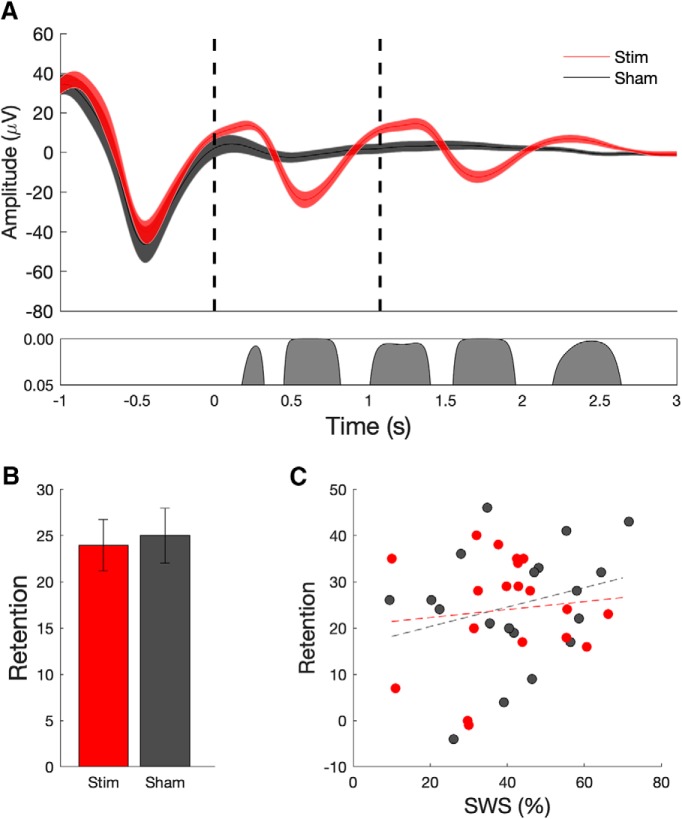
Closed-loop stimulation during overnight sleep enhances slow-wave and spindle oscillations, but does not enhance verbal memory performance. ***A***, Mean ± SEM EEG signal (at electrode Cz) averaged (across 19 subjects) time locked to the first auditory stimulus (*t* = 0 s) for the stimulation (red) and sham (black) conditions. The bottom panel indicates significant differences between conditions. Evoked responses from 19 subjects show that stimulation delivered during slow-wave UP states (red curves; dashed lines indicate onset of acoustic pulse) enhances ongoing slow-wave oscillations relative to sham stimulation (black). ***B***, Memory performance, as assessed by the word pair associates task does not exhibit a significant benefit from acoustic stimulation relative to sham (mean, SEM). ***C***, Correlation (and trend line) between the amount of SWS during the stimulation period and retention on the behavioral task. Correlations were not significant in either condition.

**Figure 5.**
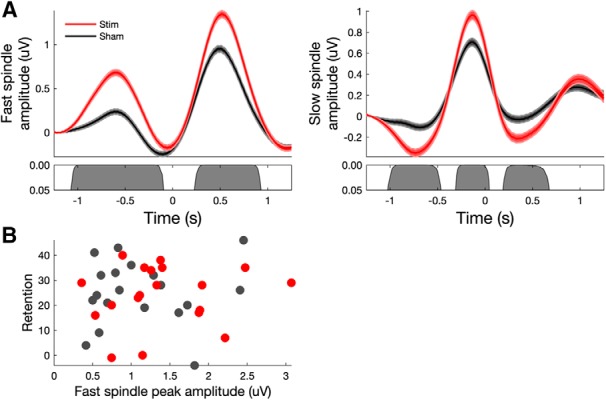
Acoustic stimulation enhances both fast- and slow-spindle amplitude but is not related to verbal memory consolidation. ***A***, Fast-spindle (12–15 Hz) and slow-spindle (9–12 Hz) amplitude at electrode Cz time locked to the negative DOWN state of all detected SO events (*t* = 0). ***B***, Scatter plot of retention versus peak fast-spindle amplitude across individuals in sham and stimulation conditions (not significant in either condition).

We found positive effects on the sleep rhythms, including significant entrainment of slow waves to the first and second auditory pulses compared with sham stimulation (Fig. 4*A*; three SO cycles in the stimulation condition compared with one SO in the sham condition). In addition, we found increased fast- and slow-spindle amplitude (time locked to all SO events over the entire night; see Materials and Methods) compared with sham stimulation (Fig. 5*A*; i.e., closed-loop acoustic stimulation produced robust physiologic entrainment of sleep rhythms). Analysis of the sleep characteristics during the 210 min stimulation period revealed no significant differences in the amount of time spent in each stage of sleep between conditions (stim vs sham; Table 5). Analysis of SO characteristics across the night of sleep showed that the total number of SO detections was similar between the stimulation and sham conditions. SO amplitude, slope, and duration were similar between stimulation and sham (Table 6). The median ISI in the stimulation condition was 10.6 s.

**Table 5.** Mean ± **SEM spent in each sleep stage during experiment 2 (overnight study)**

	Stim	Sham	*p* Value
W	1.55% (0.69)	4.11% (3.21)	0.44
N1	3.61% (1.00)	2.19% (0.64)	0.23
N2	44.50% (3.47)	39.33% (3.89)	0.33
SWS	39.69% (3.34)	40.85% (4.32)	0.83
REM	9.25% (1.35)	7.39% (1.48)	0.36
MA	1.39% (0.29)	0.89% (0.20)	0.17
Total sleep time (min)	394.4 (15.6)	398.5 (11.1)	0.83

Sleep characteristics during the 210 min stimulation period showing the average time spent in each sleep stage demonstrates that the percentage of time spent in each condition (W, wake; N1, stage 1; N2, stage 2; MA, muscle artifact) was similar during the stimulation period, suggesting that stimulation did not disrupt sleep or increase the overall time spent in NREM sleep.

**Table 6.** Characteristics of slow oscillations during overnight closed-loop stimulation (experiment 2).

	Stim	Sham	*p* Value
Number of SOs (stimulation period)	529.37 (74.71)	477.05 (60.75)	0.59
Number of SOs (entire night)	1356.37 (111.47)	1338.89 (106.62)	0.87
SO amplitude (μV)	141.93 (9.16)	128.62 (10.06)	0.24
SO slope (μV/s)	277.64 (21.97)	244.02 (20.80)	0.14
Duration (s)	1.18 (0.01)	1.14 (0.07)	0.49

Mean ± SEM number of slow oscillations (SOs identified off-line; see Materials and Methods) during SWS epochs of the stimulation period and the entire night, amplitude (negative half-wave to positive-peak), slope, and duration between stim and sham conditions.

In the stimulation condition, our subjects were able to remember an additional 23.9 word pairs (±2.79 words; postsleep − presleep performance). In the sham condition, our subjects were able to remember an average of 25 word pairs (±2.96 words). Performance was not affected by floor or ceiling effects, with subjects recalling on average 33% of the word pairs before sleep, and 53% of word pairs after sleep. Again, in contrast to the reliable physiologic changes, we did not observe an effect of acoustic stimulation on memory performance, which was measured as in the original study as the number of word pairs correctly reproduced (Fig. 4*B*; *t*_(18)_ = 0.51, *p* = 0.62). On a group level, there was no significant correlation between the amount of slow-wave sleep versus word retention (Fig. 4*C*) or fast-spindle amplitude at the peak of spindle versus word retention (Fig. 5*B*), measures previously found to be correlated (Ngo et al., 2013).

## Discussion

We confirmed that a closed-loop acoustic stimulation technique can reliably enhance slow-wave activity during NREM sleep; however, this physiologic effect did not enhance memory for either of two declarative memory tasks. We first tested a closed-loop single-pulse acoustic paradigm during nap sleep. Despite inducing additional SO spindle complexes, performance was unchanged in a visuospatial navigation or a verbal paired associates task. Because of these null behavioral findings, we performed a second study, which closely replicated the original study that reported enhanced memory using acoustic stimulation during NREM sleep (Ngo et al., 2013). As later memory recall can depend on the strength of the word associations (Payne et al., 2012), we translated the same German word pairs as used in the original demonstration study (Ngo et al., 2013) into English. Compared with the original single-blinded study, our study was better powered to detect a moderate effect size (19 vs 11 subjects) and was double-blinded (subject and experimenter were blinded). Our study also included more subjects compared with previous studies: 15 young adults in an overnight study (Leminen et al., 2017), 16 young adults in a nap study (Ong et al., 2016), and 13 older adults in an overnight study (Papalambros et al., 2017). Again, we found that closed-loop acoustic stimulation acutely augmented SO spindles, but not memory performance. Our physiologic findings resembled previous studies demonstrating that a single or paired pulse during the SO DOWN-to-UP transition can induce additional SO spindle complexes, as seen on scalp EEG measurements (Ngo et al., 2013; Ong et al., 2016; Leminen et al., 2017; Papalambros et al., 2017).

### Acoustic pulses did not disrupt sleep or spindle refractory periods

We questioned whether the acoustic pulses could have interrupted the quality of sleep, thus explaining the lack of behavioral effect. We did not find evidence that sleep quality, as represented by a blinded comparison of sleep stages between the two conditions, was different in either the nap or overnight experiments (Tables 2, 5).

We also asked whether the sound pulses could have disrupted the post-spindle refractory window, which has been hypothesized to facilitate integration of new information into long-term storage. Spindles may parse sleep into optimal windows for reactivation, interleaved with refractory periods, which separate reactivation of different memories, to prevent interference (Antony et al., 2019). During sleep, naturally occurring spindles are separated by intervals ranging from 3 to 6 s. While cued acoustic reactivation during sleep can enhance memory consolidation, presenting the cue immediately after a spindle eliminates the memory benefit (Schreiner et al., 2015; Antony et al., 2018). This refractory window may range from <1.5 s after a cued presentation (Schreiner et al., 2015) to 3 s after spindle onset (Antony et al., 2018). These intervals suggest that there is a limit to the number and timing of reactivations that can occur in a sleep period (Antony et al., 2018). We wondered whether our closed-loop acoustic stimulation was delivered too closely spaced together, potentially disrupting the refractory interval. However, we found that in the overnight stimulation condition, the median interstimulus interval was 10.6 s. Thus, the timing of our acoustic stimulus was delivered outside of the putative spindle refractory window, and therefore was unlikely to interrupt the potential reprocessing of information.

### Comparison with previous studies

For the replication study in experiment 2, our subjects’ baseline performance was better than that of the subjects in the original overnight acoustic stimulation study (Ngo et al., 2013), as measured by improvement in the number of word pairs in the sham overnight condition. In the sham condition, our subjects were able to remember an average of 25 more word pairs after sleep (SEM ± 2.96 words; postsleep − presleep performance), whereas subjects in the study by Ngo et al. (2013) were able to remember 13 more word pairs (SEM ±2.5). Improvement in the stimulation condition was comparable between studies. While our subjects did not reach ceiling or floor performance on the test, it is possible that our subject population was different from the original study, although similar eligibility criteria were used. Differences in phonological encoding between languages may also have contributed to different results. While the same word pairs were used, and thus semantics were matched across studies, differing phonological properties between languages may have facilitated improved learning and retention (Copeland and Radvansky, 2001).

Endogenous NREM sleep characteristics were also slightly different in our subject population compared with the original study. Specifically, our subjects’ peak SO frequency (mean, 0.87 ± 0.1 Hz) was slightly slower than the subjects in the original study (mean, 1.03 ± 0.1 Hz). This is unlikely due to age differences, as our subjects were comparable in age (mean age, 23.3 ± 0.9 years) compared with the original study (24.2 ± 0.9 years). This difference in SO frequency is relevant to the timing of the second (paired) acoustic pulse during the overnight study. For subjects with a slower endogenous SO frequency, a fixed 1.075 s interstimulus interval may not optimally entrain the second and third SO spindle trains. Indeed, as seen in Figure 4*A* and Table 6, evoked SO trains elicited in the stimulation condition were not as high amplitude as the ones evoked in the original study. While native SO frequency was measured during the accommodation night to determine the optimal timing of the initial acoustic stimulus, the timing of the second pulse subjects were recruited may have been optimized by considering the endogenous SO frequency of our subject population as well.

To our knowledge, there are three previous studies that demonstrate that augmenting slow waves and/or spindles through acoustic stimulation is insufficient to improve memory above sham levels (Cox et al., 2014b; Weigenand et al., 2016; Ngo et al., 2019). These studies applied acoustic stimulation with varying parameters, including closed-loop single-pulse stimulation (Cox et al., 2014b), open-loop three-pulse trains (Weigenand et al., 2016), and closed-loop seven-pulse trains at spindle frequency (Ngo et al., 2019). While SO and spindle activity was enhanced in these studies, no memory improvement was demonstrated. On the other hand, there have been three studies using variable stimulation techniques, including closed-loop five-pulse (Ong et al., 2016; Papalambros et al., 2017) and closed-loop single-pulse (Leminen et al., 2017) stimulation to increase SO spindles, which did replicate the memory effect, although with more modest effect sizes (Table 7). These diverse patterns of stimulation demonstrate that there are many ways of inducing SOs and spindles. Yet, despite enhancement of these key sleep rhythms, the behavioral effects have been mixed. Our experiments extend previous work by suggesting that enhancement of SO spindle rhythms, even through replication of acoustic and behavioral paradigms, is insufficient to augment memory. Further research is needed to systematically identify which parameters of acoustic stimulation and/or subject characteristics are necessary to provide a memory enhancement and the mechanisms by which this benefit occurs.

**Table 7.** Comparison of previous studies using acoustic stimulation to boost slow oscillations and spindle and their effects on memory

Study	Study design		Behavioral results	EEG results
Ngo et al., 2013	*N* = 11 healthy young adults (mean ± SD age, 24.2 ± 2.98 years)	Stimulation protocol	Hedges *g*_av_ = 1.07 Retention (presleep − postsleep): Stim: 22.2 ± 2.3 words Sham: 13.0 ± 2.5 words	Increase in slow-wave and spindle power
		Closed-loop Two-pulse stimulation overnight sleep (7 h)		
		Memory tests		
		120 semantically related word pairs		
Cox et al., 2014b	*N* = 12 healthy adults (age range, 18–23 years; 11 females)	Stimulation protocol	No behavioral effect observed	Increased slow-wave amplitude and spindle band power with sounds targeted at up-state
		Closed-loop One-pulse stimulation Evening nap (2 h)		
		Memory tests		
		Sound stimulus memory task		
Ong et al., 2016	*N* = 16 healthy young adults (mean ± SD age, 22 ± 1.4 years)	Stimulation protocol	Hedges *g*_av_ = 0.41Retention (presleep − postsleep):Sham: −1.72 ± 4.16 SDsStim: 0.0 ± 3.76 SDs	Increased slow-wave amplitude, theta, and fast-spindle activity
		Closed-loopFive consecutive pulsesAfternoon nap (1.5 h)		
		Memory tests		
		40 semantically related word pairs		
Weigenand et al., 2016	*N* = 26 healthy young adults (mean age, 22.2 years; age range, 18– 28 years)	Stimulation protocol	No behavioral effect observed	Increase in slow-wave and increase in spindle power with first pulse
		Open-loopThree consecutive pulse stimulationOvernight sleep (7 h)		
		Memory tests		
		120 semantically related word pairs^1^		
Leminen et al., 2017	*N* = 15 healthy adults (mean age, 30.5 years; range, 23–42 years)	Stimulation protocol	Hedges *g*_av_ = 0.65Retention (presleep – postsleep):Stim: 21.1 ± 7.7 SDsSham: 15.6 ± 8.1 SDsNo behavioral effect on face–name memory, finger tapping, or picture memory (tasks 2–4)	Increase in slow-wave and spindle power
		Closed-loopSingle-pulse stimulationOvernight sleep (7 h)		
		Memory tests		
		120 semantically related word pairs^2^ Face–name association testFinger-tapping testPicture recognition task		
Papalambros et al., 2017	*N* = 13 healthy older adults (mean age, 75.2 years; age range, 60–84 years)	Stimulation protocol	Hedges *g*_av_ = 0.77Retention (presleep – postsleep):Stim: 9.2 ± 7.93 SDsSham: 3.1 ± 6.85 SDs	Increase in slow-wave and spindle power
		Closed-loopFive-pulse, phase-locked loop stimulationOvernight sleep (8 h)		
		Memory tests		
		88 semantically related word pairs		
Ngo et al., 2019	*N* = 24 healthy young adults (mean ± SD age, 23.9 ± 3.42 years)	Stimulation protocol	No behavioral effect observed	Acute increase in slow-wave and spindle power, but no effect on total overnight slow-wave and spindle power
		Closed-loopSeven-click spindle stimulationOvernight sleep (7 h)		
		Memory tests		
		120 semantically related word pairs^1^		

For each study, the brief description of the subject pool, stimulation and memory protocols, effect size (Hedges *g*_av_) of the behavioral effect (if applicable), and overall electrophysiological findings are provided.

1 Same word lists used in Ngo et al., 2013.

2 Translated from list used in Ngo et al., 2013.

### Behavioral specificity and spindle enhancement of memory

A growing body of evidence suggests that sleep does not equally benefit all types of memory. Some evidence suggests that sleep favors learning under explicit circumstances, when subjects are aware of what they are learning (Fischer et al., 2002, 2006; Robertson et al., 2004; Korman et al., 2007), compared with implicit or unconscious learning conditions (Robertson et al., 2004; Spencer et al., 2006; Song et al., 2007). Emotional memories may be more strongly remembered after sleep compared with neutral memories (Wagner et al., 2006; Payne et al., 2008; Nishida et al., 2009; Javadi et al., 2011; Payne and Kensinger, 2011; Baran et al., 2012), although other studies did not find these effects (Sterpenich et al., 2007, 2009; Lewis et al., 2011). Sleep appears to favor the strengthening of information relevant to future goals (Cohen et al., 2005; Fischer and Born, 2009; Scullin and McDaniel, 2010; Wilhelm et al., 2011; van Dongen et al., 2012; Diekelmann et al., 2013a,b) including items that are cued on learning to be relevant to future rewards (Fischer and Born, 2009).

Because of these behavioral considerations, we explicitly instructed subjects that they would be retested at a later point in the study. Furthermore, in the second experiment, we attempted to remove forms of subtle communication of future relevance or reward by blinding both subjects and experimenter to the condition.

One general consideration is that a nonspecific stimulus was applied to enhance memory for a specific task. In reality, human subjects are exposed to a variety of stimuli during the previous day, which may possess more salience than the word pairs presented in the laboratory before sleep. Further, it is unclear how global enhancement of SO spindles relates to domain-specific stabilization of memory traces, supported by local spindle activity. SO spindles seen on EEG reflect a global summated signal, whereas magnetoencephalogram (MEG) and electrocorticography (ECoG) studies of local field potentials during sleep suggest that spindles occur on both local and global scales (Andrillon et al., 2011; Antony et al., 2019). Previous attempts to enhance specific memories include targeted memory reactivation, in which odors or sounds associated with learned objects during the learning phase were later played during NREM sleep to reactivate specific learned stimuli (Rasch et al., 2007; Rudoy et al., 2009; Antony et al., 2012; Rihm et al., 2014; Schreiner and Rasch, 2015; Cairney et al., 2016).

### Induced spindles may be insufficient to augment memory consolidation

By driving additional SO spindle complexes via acoustic stimulation, a reasonable assumption is that the efficiency of information transfer from hippocampus to neocortex is enhanced, through indirectly increasing nested SPW-R events. However, despite several reports emphasizing the role of spindles in memory reinstatement and consolidation during sleep, our findings challenge the causal role of artificially induced sleep spindles in reactivation.

Although previous experiments have demonstrated a tripartite relationship between sleep spindles and hippocampal SPW-Rs, the coupling probability between spindles and SPW-Rs is relatively low (Siapas and Wilson, 1998; Mölle et al., 2002, 2006; Sirota et al., 2003; Clemens et al., 2007, 2011; Gelinas et al., 2016; Song et al., 2017). A possible explanation for the lack of memory enhancement by acoustic stimulation is that stimulation-induced and spontaneously occurring spindles are not identical, and artificial spindles may not efficiently entrain hippocampal SPW-Rs. We found evidence of a global system refractoriness, which may limit the total number of memory reactivations across a period of sleep (Ngo et al., 2015). Similar to previous studies, we found that, despite evoking additional SO spindle complexes in trains, the time spent in NREM stages 2 and 3 of sleep was similar between stimulation and sham conditions for both experiments (Ngo et al., 2019; Tables 2, 5). Likewise, the overall number of SO events during the stimulation and sham conditions in both experiments was similar (Tables 3, 6).

Further studies are needed to demonstrate that artificially induced spindles are coupled with SPW-Rs and memory reactivation. Reactivation of the learned information in sleep EEG should be detected, and the decoded replay should be related to sleep spindles (Bergmann et al., 2012; Schönauer et al., 2017). Source localization of hippocampal SPW-Rs using high-density MEG in conjunction with the detection of spontaneous and induced spindles in human subjects may be measured in humans (Liu et al., 2019).

### Conclusions

Despite the belief that coordinated slow oscillation, spindle, and sharp-wave ripples are cardinal brain rhythms supporting sleep-dependent memory consolidation, inducing SO spindle trains through closed-loop acoustic stimulation was insufficient to enhance declarative memory on virtual navigation or verbal paired associates tasks. From a physiologic perspective, a global system refractoriness to increasing the number of SO spindles across the entire period of sleep may limit the number of additional SPW-R reactivations facilitated by spindle events. Or if SPW-Rs are increased, these reactivations may differ from those tested and measured in the laboratory. Finally, differences in subject populations may account for our inability to replicate previously reported beneficial memory effects.

## Synthesis

Reviewing Editor: Bradley Postle, University of Wisconsin Decisions are customarily a result of the Reviewing Editor and the peer reviewers coming together and discussing their recommendations until a consensus is reached. When revisions are invited, a fact-based synthesis statement explaining their decision and outlining what is needed to prepare a revision will be listed below. The following reviewer(s) agreed to reveal their identity: Björn Rasch, Flavio Frohlich. Note: If this manuscript was transferred from JNeurosci and a decision was made to accept the manuscript without peer review, a brief statement to this effect will instead be what is listed below.

Here is the full text of the two reviews:

R#1 - The authors replicated the effects of closed-loop acoustic stimulation on slow-oscillations and sleep spindles in an afternoon nap and nighttime sleep. In spite of these physiological changes, they were not able to reproduce the beneficial effects of closed-loop acoustic stimulation during sleep on declarative memory performance.

The topic of the paper is highly relevant and important. The authors can be congratulated that they not only studied and report the effects of the nap experiment, but also aimed at closely replicating the effects of closed-loop stimulation during nighttime sleep on declarative memory consolidation. Taken together, these two null effects are highly relevant for our understanding of the influences of close-loop acoustic stimulation on sleep-associated declarative memory consolidation. I fully support publication of this well-conducted studies and well-written paper. I have only some minor comments:

Minor comments:

Abstract: Please indicate that only 12 participants were included in the final analysis of the nap study.

Page 2, line 104 and 108: Please provide more details for the power calculation, i.e., indicate the effect size and the assumed correlation between measurements. Note that Cohen defined d only for independent measurements.

Page 8, line 71: Please provide more details with respect to sleep spindles. Did the number of sleep spindles change? Was the amplitude affected ? Difference between fast and slow spindles? Correlation with retention scores? Please be more consistent in your analysis and reporting of outcome parameters for Exp. 1 and Exp. 2.

Table 7, Ong et al., 2016: There is an error in the retention score: Stim and Sham are exchanged, please correct.

R#2 - I have reviewed many papers and never before recommended acceptance as is. This is a very important and very clear manuscript. Congratulations!

## Author Response

Dear Dr. Postle,

We would like to thank the reviewers for their thoughtful comments on our manuscript, Closed-loop acoustic stimulation enhances sleep oscillations but not memory performance (eN-TNRS-0306-19X). Below, please find our responses to the reviewers' comments.

Anli Liu

-----------

R#1 - The authors replicated the effects of closed-loop acoustic stimulation on slow-oscillations and sleep spindles in an afternoon nap and nighttime sleep. In spite of these physiological changes, they were not able to reproduce the beneficial effects of closed-loop acoustic stimulation during sleep on declarative memory performance.

The topic of the paper is highly relevant and important. The authors can be congratulated that they not only studied and report the effects of the nap experiment, but also aimed at closely replicating the effects of closed-loop stimulation during nighttime sleep on declarative memory consolidation. Taken together, these two null effects are highly relevant for our understanding of the influences of close-loop acoustic stimulation on sleep-associated declarative memory consolidation. I fully support publication of this well-conducted studies and well-written paper. I have only some minor comments:

Minor comments:

Abstract: Please indicate that only 12 participants were included in the final analysis of the nap study.

Thank you for pointing out this discrepancy. We have changed the number of subjects who completed Experiment 1 to twelve (12) in the abstract.

Page 2, line 104 and 108: Please provide more details for the power calculation, i.e., indicate the effect size and the assumed correlation between measurements. Note that Cohen defined d only for independent measurements.

We thank the reviewer for pointing this out. To account for the correlation between measures, we have decided to report Hedges gav, a more suitable measure of effect size for within-subjects designs. This measure uses the average across-condition standard deviation, dz, and correction for the number of within-subject comparisons (e.g. N). We have updated Table 7 to report Hedges gav and included a description of this analysis in the Methods.

Page 8, line 71: Please provide more details with respect to sleep spindles. Did the number of sleep spindles change? Was the amplitude affected ? Difference between fast and slow spindles? Correlation with retention scores? Please be more consistent in your analysis and reporting of outcome parameters for Exp. 1 and Exp. 2.

In order to make the analyses comparable for Experiments 1 and 2, we added a new figure which presents fast and slow spindle band changes time-locked to the SO downstate observed for Experiment 1 nap study (Figure 3).

However, we have chosen not to compare the number of spindles between stimulation and sham conditions. Spindle detection algorithms can yield highly variable spindle counts, which would confuse any direct comparison between this study and previously published results. Therefore, we chose to replicate the analysis performed in the original study (Ngo. Et al, 2013), to compare change in power of the SO-locked fast and slow spindles between conditions.

Table 7, Ong et al., 2016: There is an error in the retention score: Stim and Sham are exchanged, please correct.

Thank you for pointing out this discrepancy. We have changed this in Table 7.

R#2 - I have reviewed many papers and never before recommended acceptance as is. This is a very important and very clear manuscript. Congratulations!

Thank you for your encouragement.
